# Initial chest radiographs and artificial intelligence (AI) predict clinical outcomes in COVID-19 patients: analysis of 697 Italian patients

**DOI:** 10.1007/s00330-020-07269-8

**Published:** 2020-09-18

**Authors:** Junaid Mushtaq, Renato Pennella, Salvatore Lavalle, Anna Colarieti, Stephanie Steidler, Carlo M. A. Martinenghi, Diego Palumbo, Antonio Esposito, Patrizia Rovere-Querini, Moreno Tresoldi, Giovanni Landoni, Fabio Ciceri, Alberto Zangrillo, Francesco De Cobelli

**Affiliations:** 1 Clinical and Experimental Radiology Unit, Experimental Imaging Center, IRCCS San Raffaele Scientific Institute, Milan, Italy; 2 Faculty of Medicine and Surgery, Vita-Salute San Raffaele University, Via Olgettina 58, Milan, Italy; 3 Department of Internal Medicine, IRCCS San Raffaele Scientific Institute, Milan, Italy; 4 Unit of General Medicine and Advanced Care, IRCCS San Raffaele Scientific Institute, Milan, Italy; 5 Department of Anesthesia and Intensive Care, IRCCS San Raffaele Scientific Institute, Milan, Italy; 6 Hematology and Bone Marrow Transplantation, IRCCS San Raffaele Scientific Institute, Milan, Italy

**Keywords:** Radiography, Artificial intelligence, COVID-19, Severe acute respiratory syndrome, Prognosis

## Abstract

**Objective:**

To evaluate whether the initial chest X-ray (CXR) severity assessed by an AI system may have prognostic utility in patients with COVID-19.

**Methods:**

This retrospective single-center study included adult patients presenting to the emergency department (ED) between February 25 and April 9, 2020, with SARS-CoV-2 infection confirmed on real-time reverse transcriptase polymerase chain reaction (RT-PCR). Initial CXRs obtained on ED presentation were evaluated by a deep learning artificial intelligence (AI) system and compared with the Radiographic Assessment of Lung Edema (RALE) score, calculated by two experienced radiologists. Death and critical COVID-19 (admission to intensive care unit (ICU) or deaths occurring before ICU admission) were identified as clinical outcomes. Independent predictors of adverse outcomes were evaluated by multivariate analyses.

**Results:**

Six hundred ninety-seven 697 patients were included in the study: 465 males (66.7%), median age of 62 years (IQR 52–75). Multivariate analyses adjusting for demographics and comorbidities showed that an AI system-based score ≥ 30 on the initial CXR was an independent predictor both for mortality (HR 2.60 (95% CI 1.69 − 3.99; *p* < 0.001)) and critical COVID-19 (HR 3.40 (95% CI 2.35–4.94; *p* < 0.001)). Other independent predictors were RALE score, older age, male sex, coronary artery disease, COPD, and neurodegenerative disease.

**Conclusion:**

AI- and radiologist-assessed disease severity scores on CXRs obtained on ED presentation were independent and comparable predictors of adverse outcomes in patients with COVID-19.

**Trial registration:**

ClinicalTrials.gov NCT04318366 (https://clinicaltrials.gov/ct2/show/NCT04318366).

**Key Points:**

*• AI system–based score ≥ 30 and a RALE score ≥ 12 at CXRs performed at ED presentation are independent and comparable predictors of death and/or ICU admission in COVID-19 patients.*

*• Other independent predictors are older age, male sex, coronary artery disease, COPD, and neurodegenerative disease.*

*• The comparable performance of the AI system in relation to a radiologist-assessed score in predicting adverse outcomes may represent a game-changer in resource-constrained settings.*

## Introduction

A novel coronavirus, severe acute respiratory syndrome coronavirus 2 (SARS-CoV-2), that causes the coronavirus disease 2019 (COVID-19) was first identified in December 2019 in Wuhan, China [1].

Severe complications of COVID-19 include severe pneumonia, acute respiratory distress syndrome, multiple organ failure, and death [2]. Since initial detection, the virus has rapidly spread across the world, infecting more than 14 million people and killing over 590,000 [3].

As the COVID-19 pandemic keeps overwhelming healthcare systems worldwide, a prompt large public health surveillance and response program is needed. Real-time reverse transcriptase polymerase chain reaction (RT-PCR) remains the reference standard for diagnosis, but its high false-negative rate, limited testing capacity, and long turnaround times hinder its effectiveness most likely contributing to the spread of the infection within communities [4].

In this scenario, the role of imaging with chest X-ray (CXR) and chest computed tomography (CT) may become fundamental in quickly providing results that can guide in terms of triage and clinical management [5].

The few currently available reports on the use of CXR for COVID-19 diagnosis seem to point towards the fact that this imaging modality lacks sensitivity in identifying some of the otherwise evident findings visible on CT [6–9]. However, during the SARS coronavirus outbreak in 2003, CXR radiographic findings were found to be associated with worse clinical outcomes [10–12], similar to what has been recently reported in COVID-19 infection in young and middle-aged adults [13].

In resource-constrained settings with large throughputs to handle, such as with the COVID-19 pandemic, artificial intelligence (AI) may help expedite reading times, thus becoming an important asset in the clinical management of these patients. An AI system for the detection of COVID-19 was recently shown to be able to identify COVID-19 pneumonia on CXR with performance comparable to six independent radiologists [14]. However, the role of AI on CXR as a prognostic tool has not yet been evaluated in COVID-19 patients.

Taken together, the high immediate and widespread availability of CXR, the reduced risk of cross-infection and low-cost compared with CT imaging, and the potential to become a first-line triage tool are worth further investigation to increase our understanding of the predictive role of radiographic features in COVID-19 [5].

In this context, our study aims to identify and quantify COVID-19 CXR findings, assess the relationship between initial CXR severity and clinical outcomes, and evaluate the use of an AI system as an initial prognostic tool in COVID-19.

## Methods

### Patients

This series is part of the COVID-19 Institutional clinical-biological cohort assessing patients with COVID-19 (COVID-BioB, ClinicalTrials.gov NCT04318366) at a 1350-bed tertiary care academic hospital in Milan, Italy. The study was approved by the ethics committee (EC) (protocol number 34/INT/2020). All procedures were conducted in agreement with the 1964 Helsinki declaration and its later amendments; informed consent was collected from all patients according to the EC guidelines.

**Fig. 1:**
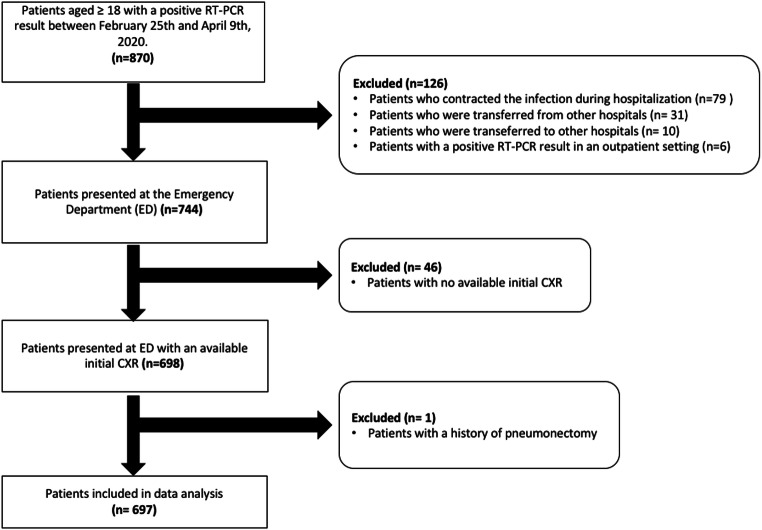
Flow diagram of our retrospective single-center cohort study

All consecutive patients aged ≥ 18 years, admitted to the Institution’s Emergency Department (ED) with a positive RT-PCR nasopharyngeal swab between February 25 and April 9, 2020, were initially considered. Patients with a CXR obtained on presentation were included in the study. Exclusion criteria were patients who acquired infection during hospitalization, those transferred to the institution from other hospitals or later transferred to other hospitals, and those with positive RT-PCR as outpatients. A complete exclusion flow diagram is provided in Fig. 1.

### Clinical data collection

All prospectively collected clinical data were retrospectively extracted from the study’s dedicated electronic database.

The time-to-event for clinical outcomes, i.e., death, admission to intensive care unit (ICU), and discharge, was calculated from the date of hospital admission to the date of the event; follow-up was right-censored on May 5, 2020.

Clinical outcomes categories were defined as (i) death (primary) and (ii) critical COVID-19, which included patients admitted to ICU and deaths occurring before ICU admission.

### Imaging data collection and evaluation

Conventional chest X-ray (CXR) images were acquired in the posteroanterior (PA) or in the anteroposterior (AP) projection for patients not able to stand. All AP projection images were acquired with portable X-ray machines with patients in a supine position or sitting up.

Radiographs obtained on ED presentation were reviewed by two radiologists (F.D.C. and C.M.A.M., respectively, with 30 years and 24 years of experience in thoracic imaging); agreement was obtained by consensus. To minimize bias, reviewers had no knowledge of clinical data other than COVID-19 positivity.

The following radiographic findings were evaluated: hazy opacities, consolidation, hilar enlargement, and pleural effusion [15]. Lung opacities’ distribution was assessed and categorized as follows: peripheral/peri-hilar predominance, upper/lower quadrant predominance, or no predominance and bilateral or unilateral involvement.

The severity of lung involvement, on all baseline CXRs, was quantified by a deep learning artificial intelligence (AI) system (qXR v2.1 c2, Qure.ai Technologies) and compared with a radiologist-assessed score.

qXR is a CE-certified deep learning AI system based on a set of convolutional neural networks (CNNs) trained to detect a number of specific abnormalities on frontal CXRs (blunting of costophrenic angle, cardiomegaly, cavitation, consolidation, fibrosis, hilar enlargement, nodules, opacities and pleural effusion). The specific architectures that form the basic blocks in the systems and detect individual abnormalities are versions of residual neural networks (ResNets) with squeeze-excitation modules with abnormality-specific modifications. The AI system identifies normal CXRs and detects and localizes suspect abnormalities providing results in terms of percentage of involvement and, if necessary, reports the pre-defined tags.

The algorithm was trained on a set of 2.3 million CXRs collected from different centers in different geographical locations [16]. Two different datasets (respectively consisting of more than 89,000 and 2000 distinct CXRs) were used for validation and another set of images for algorithm development. A validated natural language processing algorithm identified the defined abnormalities in the original radiology reports, in the largest dataset, which were considered the gold standard. The developers report that the algorithm, using the radiologists’ assessment as the gold standard, achieved an area-under-the-curve (AUC) for the detection of the specific abnormalities varying from 0.89 to 0.98; notably, AUCs of 0.95 (95% CI 0.92–0.98) for consolidation and 0.94 (95% CI 0.93–0.96) for opacities [16]. The algorithm was additionally tuned with recent images from COVID-19-positive and COVID-19-negative patients [17].

**Fig. 2:**
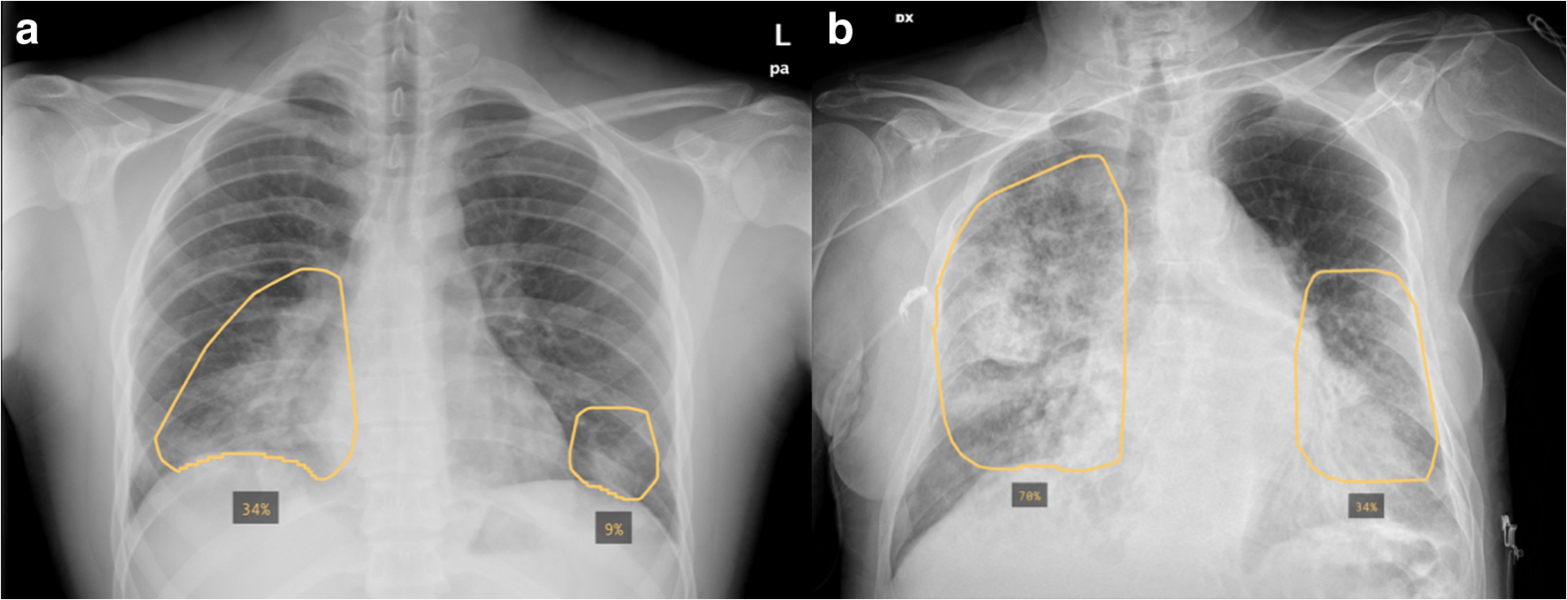
Examples of the AI system (qXR v2.1 c2, Qure.ai Technologies) analysis overlay on initial CXRs of two patients in our cohort showing the percentage of pixels involved by opacity or consolidation for each lung. **a** Posteroanterior CXR of an 18-year-old male (34% right lung; 9% left lung; Qure AI score [(34 + 9)/2] = 21.5). **b** Anteroposterior CXR of an 81-year-old male (70% right lung; 34% left lung; Qure AI score [(70 + 34)/2] = 52)

For the purpose of our study, the software output was personalized to only report the extent of consolidation and lung opacities. The severity of the lung involvement was calculated by the AI system as the percentage of pixels involved by opacity or consolidation for each lung (cutoff 3%). The average of the two values ((percentage of right lung involvement + percentage of left lung involvement)/2), Qure AI “score,” was then obtained to reflect total lung involvement (minimum score 0 = no lung involvement; maximum score 100 = complete opacification/consolidation of both lungs) as described in Fig. 2.

The same CXRs were then evaluated by the radiologists using the Radiographic Assessment of Lung Edema (RALE) score to quantify the severity of lung abnormalities [18]. Each CXR was divided into quadrants and each quadrant was assigned a score by a radiologist which described the (i) extent of opacities (0–4; absence, < 25%, 25–50%, 50–75%, and > 75% involvement) and (ii) density of opacity (1–3; hazy, moderate, or dense). The final score (maximum 48) was obtained by summing the product of the consolidation and density scores for each of the four quadrants.

### Statistical analysis

Patients’ characteristics were assessed with standard descriptive statistics. Frequencies presented as percentages were used to express categorical values; median values with respective interquartile ranges (IQR) were used for continuous variables. Imputation for missing data was not performed.

To evaluate the sensitivity of the initial CXR, radiological scores of > 0 were interpreted as positive.

The correlation between the two radiological scores was assessed by Kendall’s rank correlation test.

Baseline CXR lung opacity characteristics and radiological scores of patients with symptoms suggestive for COVID-19 for < 7 days or ≥ 7 days at ED presentation were compared using the chi-square test and Mann-Whitney *U* test, respectively; the cutoff at 7 days was selected as the median value.

The ability of the AI calculated total lung involvement and the radiologist-assessed RALE score to predict mortality and critical COVID-19 was determined by the area-under-the-curve (AUC) of receiver operating characteristics (ROC) curves. The optimal cutoff values were determined on the highest Youden index value for the primary outcome (mortality) and were used to estimate Kaplan-Meier curves for survival and ICU-free survival, which were compared by the log-rank test.

A Cox proportional hazard model including sex and age (model 1) was used to evaluate the association between radiological scores and clinical outcomes. A second more comprehensive Cox proportional hazard model (model 2) which in addition to model 1 variables included important comorbidities, or known risk factors, was also used. Effect estimates were reported as hazard ratios (HRs) with 95% confidence intervals (CIs).

The correlation between the RALE or Qure AI score and clinical signs (considered temperature and PaO2/FiO2 ratio) were evaluated using two-tailed Pearson’s correlation or Kendall’s tau based on the distribution of the variables.

Two-tailed tests were performed, and a *p* value of < 0.05 was considered statistically significant.

Statistical analyses were performed using SPSS 26 (SPSS Inc./IBM) and SAS version 9.4.

## Results

### Clinical data

Six hundred and ninety-seven (697) patients were included in the study (Table 1): 465 males (66.7%), with a median age of 62 years (IQR 52–75). The most frequent comorbidity observed was hypertension (295 patients; 42.3%) followed by diabetes (117 patients; 16.8%), coronary artery disease (86; 12.3%), chronic kidney disease (55; 7.9%), active neoplasms (38; 5.5%), chronic obstructive pulmonary disease (COPD) (34; 4.9%), and neurodegenerative disease (33; 4.7%). Comorbidity data was missing for 15 patients (2.1%).

**Table 1:** Characteristics at enrolment in 697 Italian patients with COVID-19 at emergency department (ED) presentation between February 25 and April 9, 2020

Characteristic	
*N*	697
Age, median (IQR)	62 (52–75)
Sex, *n* (%)	
M –	465 (66.7)
F	232 (33.3)
Days since symptoms onset, median (IQR)	7 (4–10)
Temperature °C, median (IQR)	37.9 (37.0–38.5)
PaO2/FiO2, median (IQR)	290 (214–337)
Negative RT-PCR at admission, *n* (%)	45 (6.5)
Comorbidities, *n* (%)	
Hypertension	295 (42.3)
Diabetes	117 (16.8)
Coronary artery disease	86 (12.3)
Chronic kidney disease	55 (7.9)
Neoplastic disease	38 (5.5)
Chronic obstructive pulmonary disease	34 (4.9)
Neurodegenerative disease	33 (4.7)

*IQR*, interquartile range; *RT-PCR*, real-time reverse transcriptase polymerase chain reaction

At ED presentation, the median body temperature was 37.9 °C (IQR 37.0–38.5) and median PaO2/FiO2 was 290 (IQR 214–337). Temperature and PaO2/FiO2 values were missing for 53 patients (7.6%) and 90 patients (12.9%), respectively. The median time from symptom onset was 7 days (IQR 4–10). Symptom onset data was missing for 38 patients (5.5%). Forty-five (45) patients (45/697, 6.5%) were negative at their first RT-PCR swab.

At the last observation cutoff date, 492 patients (70.6%) had been discharged (of which 104 immediately at the ED), 80 patients (11.5%) had been admitted to ICU, 72 patients (10.3%) were still hospitalized, and a total of 133 died (overall 19.1%; 34 in ICU and 99 inward). Details are available in Table 2.

**Table 2:** Clinical outcomes of 697 Italian patients with COVID-19 enrolled between February 25 and April 9, 2020; follow-up was right-censored on May 5, 2020

Outcome	ICU admission		Total
	No	Yes	
Discharged	474	18	492
Death	99	34	133
Still hospitalized	44	28	72
Total	617	80	697

*ICU*, intensive care unit

### CXR image analysis

Five hundred and ninety-four (594, 85.2%) CXRs were acquired in the AP projection (supine position (*n* = 493) or sitting up (*n* = 101); the remaining 103 (14.8%) radiographs were acquired in the standard PA projection. With respect to opacity types and distribution, consolidation was present in 458 patients (65.7%); 144 patients (20.7%) had hazy opacities only; bilateral involvement was seen in 517 patients (74.2%); and 89 patients (12.8%) had only unilateral involvement.

Opacity predominance was peripheral in 325 patients (46.6%) and peri-hilar in 70 patients (10.0%); lower quadrant predominance was observed in 176 patients (25.3%) while only 46 (6.6%) had an upper quadrant predominance; 189 patients (27.1%) did not show any predominance. Hilar enlargement was found in 150 patients (21.5%), while only 45 (6.5%) showed pleural effusion.

Median Qure AI and RALE scores for pulmonary involvement were respectively 29 (IQR 11–44.5) and 6 (IQR 3–13). Kendall’s rank test showed a linear correlation between the two radiological scores (*r* = 0.630, *p* < 0.0001). Qure AI score was missing for 11 patients (1.6%) due to image-transfer technical issues.

The AI system reported no involvement in 140 patients (20.4%) while the RALE score reported negative CXRs (RALE score = 0) in 91 patients (13.1%); therefore, the sensitivities were respectively 79.6% and 86.9%. There were no patients with a Qure AI score in the lower quartile (Q1, score < 11) and a RALE score in the upper quartile (Q4, score > 13). There were, although, only two cases with a RALE score in the lower quartile (0 and 3) but a Qure AI score in the upper quartile (> 44; respectively 46 and 44.5). The first case was due to the presence of dense breast tissue which was interpreted by the AI system as an increased density of the underlying lung. The second case was a patient with a large unilateral pleural effusion that was interpreted by the AI system as an extensive parenchymal consolidation.

Of the 45 patients with a negative first RT-PCR at ED presentation, 8 were reported as no involvement by the AI system and 5 were scored negative by radiologists’ assessment (RALE score = 0).

The Mann-Whitney *U* test showed that the two radiological scores were significantly higher in patients with symptoms for ≥ 7 days at ED presentation compared with those symptomatic for less than 7 days (*p* = 0.031 for Qure AI score and *p* = 0.014 for RALE score). Peripheral predominance was the only radiographic finding significantly different between the two groups (*p* = 0.001) (Table 3).

**Table 3:** Radiological scores and radiographic findings of CXRs at emergency department (ED) presentation in relation to time from symptoms onset (*n* = 659)

Characteristic	Time from symptoms onset		*p* value
	< 7 days (*n* = 294)	≥ 7 days (*n* = 365)	
Radiological score, median (IQR)			
Qure AI score	25 (0–44)	31 (15–44.5)	0.031
RALE score	5 (2–12)	6 (3–13)	0.014
Type of opacity, *n* (%)			
Consolidation	182 (61.9)	249 (68.2)	0.090
Hazy opacities	64 (21.8)	75 (20.5)	0.703
Opacities’ distribution predominance, *n* (%)			
Peripheral	117 (39.8)	193 (52.9)	0.001
Peri-hilar	33 (11.2)	30 (8.2)	0.192
Upper quadrants	17 (5.8)	24 (6.6)	0.675
Lower quadrants	70 (23.8)	95 (26.0)	0.514
Hilar enlargement, *n* (%)	61 (20.7)	77 (21.1)	0.913
Pleural effusion, *n* (%)	24 (8.2)	19 (5.2)	0.126

*IQR*, interquartile range; *RALE*, Radiographic Assessment of Lung Edema

### Clinical outcomes

Regarding the ability of the two scores to predict outcomes, the optimal cutoffs identified in the ROC curves analysis were 30 for Qure AI score (sensitivity 76.9% and specificity 58.8%) and 12 for RALE score (sensitivity 57.9% and specificity 77.0%). For mortality, the AUCs were 0.66 (Qure AI) and 0.67 (RALE); for critical COVID-19, the AUCs were 0.77 (Qure AI) and 0.75 (RALE).

**Fig. 3:**
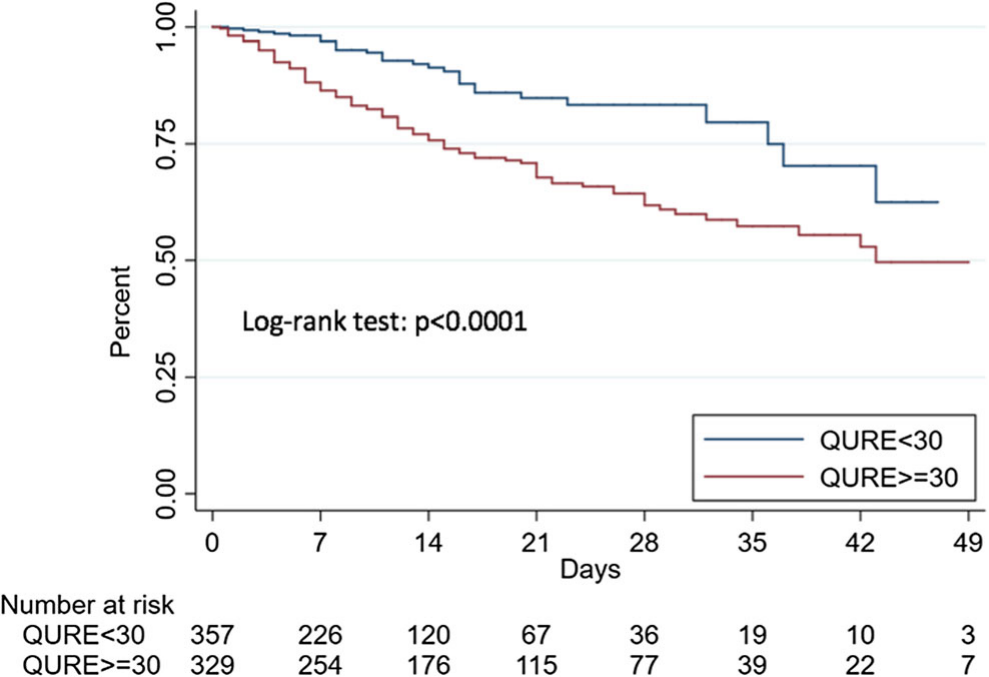
Kaplan-Meier estimates of survival according to Qure AI score optimal cutoff

**Fig. 4:**
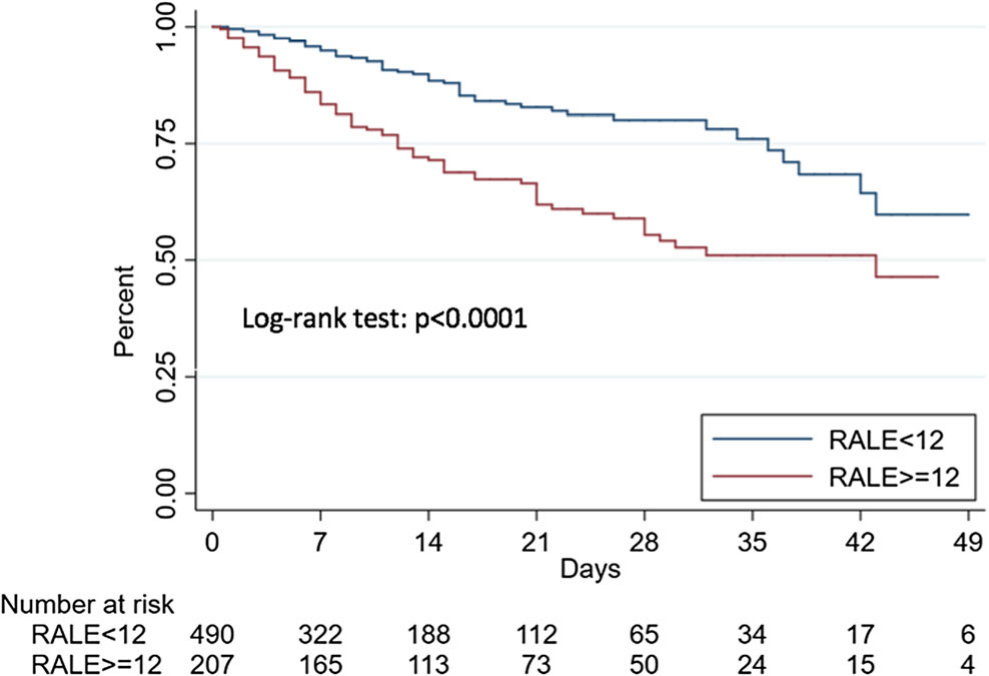
Kaplan-Meier estimates of survival according to the Radiographic Assessment of Lung Edema (RALE) score optimal cutoff

**Fig. 5:**
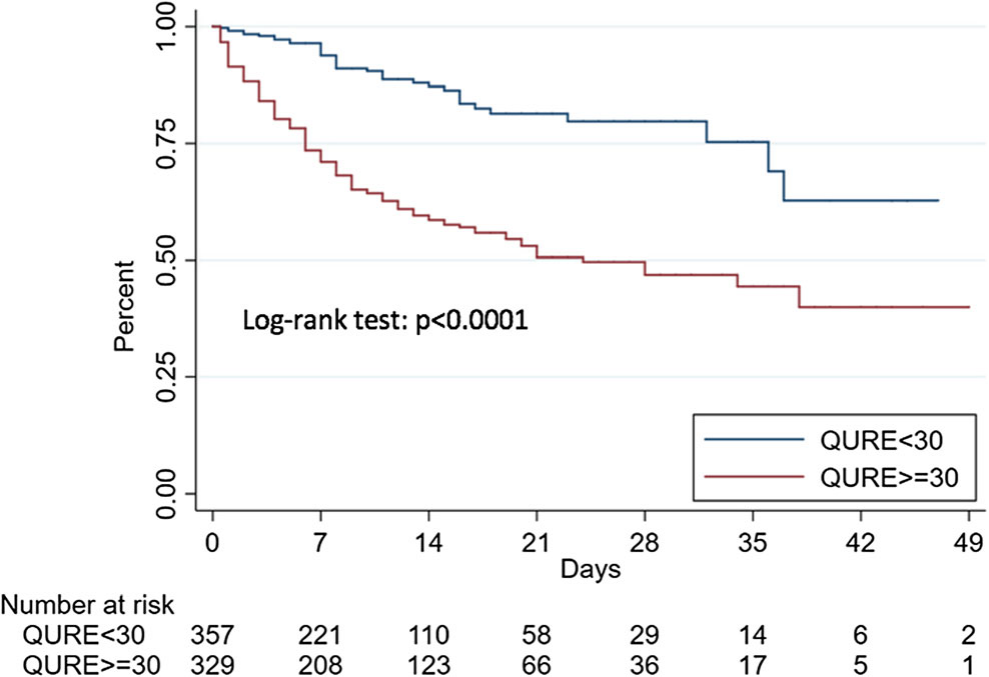
Kaplan-Meier estimates of ICU-free survival according to the Qure AI score optimal cutoff

**Fig. 6:**
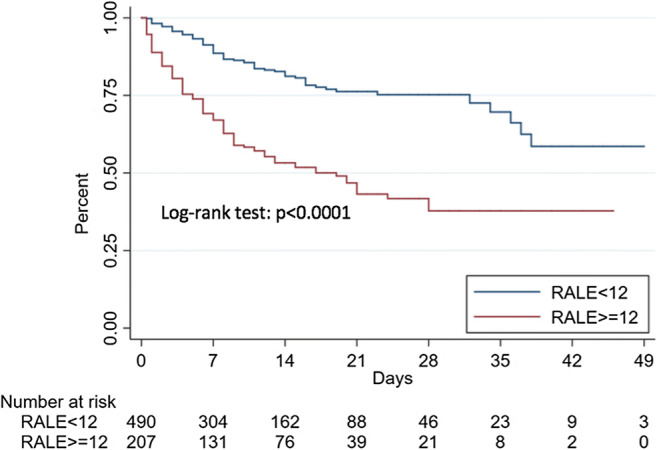
Kaplan-Meier estimates of ICU-free survival according to the Radiographic Assessment of Lung Edema (RALE) score optimal cutoff

Kaplan-Meier curves for survival and ICU-free survival estimated for the identified cutoffs for the two radiological scores were significantly different (log-rank test: *p* < 0.0001) (Figs. 3, 4, 5, and 6).

A Qure AI score of ≥ 30 was found to be an independent predictor in both models used for mortality (HR 2.45 (95% CI 1.61–3.74; *p* < 0.001) for model 1 and HR 2.60 (95% CI 1.69–3.99; *p* < 0.001) for model 2) and critical COVID-19 (HR 3.39 (95% CI 2.34–4.91; *p* < 0.001) for model 1 and HR 3.40 (95% CI 2.35–4.94; *p* < 0.001) for model 2). A RALE score ≥ 12 was also an independent predictor in both models used for mortality (HR 2.34 (95% CI 1.64–3.33; *p* < 0.001) for model 1 and HR 2.35 (95% CI 1.63–3.39; *p* < 0.001) for model 2) and critical COVID-19 (HR 2.86 (95% CI 2.11–3.88; *p* < 0.001) for model 1 and HR 2.87 (95% CI 2.10–3.91; *p* < 0.001) for model 2).

Temperature at ED presentation was not correlated to RALE or Qure AI score. At ED presentation, COVID-19 patients had a significant correlation between higher baseline scores and lower PaO2/FiO2 (RALE score − 0.353; *p* < 0.001 (Kendall’s tau) and Qure AI score − 0.476; *p* < 0.001 (Pearson’s test)).

Full results for other independent predictors are reported in Table 4.

**Table 4:** Multivariate hazard ratios (HR) of mortality and critical COVID-19*, and corresponding 95% confidence intervals (CIs), according to different radiological scores at enrolment in 697 Italian patients with COVID-19

Characteristic	Mortality (*n* = 133)				Critical COVID-19* (*n* = 179)			
	Multivariate Model 1 HR (95% CI) ^a^	*p* value	Multivariate Model 2 HR (95% CI) ^b^	*p* value	Multivariate Model 1 HR (95% CI) ^a^	*p* value	Multivariate Model 2 HR (95% CI) ^b^	*p* value
Age, for an increase of 1 year	1.05 (1.04–1.07)	< 0.001	1.03 (1.01–1.05)	< 0.001	1.02 (1.01–1.04)	< 0.001	1.01 (1.00–1.02)	0.13
Sex								
Male	1 (reference)		1 (reference)		1 (reference)		1 (reference)	
Female	0.90 (0.62–1.31)	0.59	1.03 (0.70–1.52)	0.88	0.67 (0.47–0.94)	0.02	0.70 (0.50–0.99)	0.046
Hypertension	-		1.48 (0.98–2.23)	0.06	-		1.21 (0.86–1.70)	0.27
Coronary artery disease	-		1.91 (1.28–2.85)	0.001	-		1.62 (1.12–2.35)	0.01
Diabetes	-		1.39 (0.93–2.06)	0.11	-		1.34 (0.94–1.92)	0.11
Chronic obstructive pulmonary disease	-		2.29 (1.38–3.80)	0.001	-		1.63 (1.00–2.68)	0.052
Chronic kidney disease	-		1.18 (0.78–1.78)	0.43	-		1.18 (0.81–1.74)	0.38
Neoplastic disease	-		1.55 (0.94–2.57)	0.09	-		1.04 (0.63–1.71)	0.89
Neurogenerative disease	-		2.40 (1.39–4.13)	0.002	-		2.07 (1.24–3.45)	0.006
QURE AI score								
< 30	1 (reference)		1 (reference)		1 (reference)		1 (reference)	
≥ 30	2.45 (1.61–3.74)	< 0.001	2.60 (1.69–3.99)	< 0.001	3.39 (2.34–4.91)	< 0.001	3.40 (2.35–4.94)	< 0.001
Continuous HR, increase of 1 SD (= 21.0)	1.63 (1.35–1.98)	< 0.001	1.61 (1.32–1.96)	< 0.001	2.16 (1.81–2.57)	< 0.001	2.13 (1.79–2.55)	< 0.001
RALE score								
< 12	1 (reference)		1 (reference)		1 (reference)		1 (reference)	
≥ 12	2.34 (1.64–3.33)	< 0.001	2.35 (1.63–3.39)	< 0.001	2.86 (2.11–3.88)	< 0.001	2.87 (2.10–3.91)	< 0.001
Continuous HR, increase of 1 SD (=8.7)	1.39 (1.21–1.59)	< 0.001	1.37 (1.19–1.58)	< 0.001	1.65 (1.47–1.86)	< 0.001	1.72 (1.51–1.95)	< 0.001

*RALE*, Radiographic Assessment of Lung Edema; *ICU*, intensive care unit; *HR*, hazard ratio; *CI*, confidence interval

*Patients admitted to ICU or deaths occurring before ICU admission

^a^Computed using a multivariate Cox regression model, including terms for sex and age

^b^Computed using a multivariate Cox regression model, including terms for sex, age, and comorbidities

## Discussion

Current COVID-19 radiological literature is dominated by CT and a limited number of reports describe the role of CXRs [6–9]. CT was proposed as a first-line investigation at the start of this pandemic; however, this approach has some limitations [19]. In fact, not only it increases the risk of transmission to healthcare workers and other patients, but also the necessary decontamination procedures required after scanning COVID-19 patients could obviously disrupt radiological service availability in a setting where a dedicated COVID-19 CT scanner is not available. Thus, the Fleischner Society Consensus and the American College of Radiology caution towards this approach and the latter suggests the use of CXR to minimize the risk of cross-infection [5, 20–22]. Keeping all this in consideration, and the fact that CXR is widely available and already routinely obtained in the ED, improving our understanding of the role of COVID-19 CXR radiographic features is mandatory.

In our cohort, radiographic features were consistent with those of other reports; the distribution of lung opacities (consolidation and hazy opacities) was typically bilateral, peripheral, and basilar with limited cases of pleural effusion [6, 8, 13].

To evaluate the ability of a deep learning AI-based system (qXR v2.1 c2, Qure.ai Technologies) to predict adverse outcomes in COVID-19 patients, we compared its performance with the RALE score, a radiographic score with an excellent inter-observer agreement that has been validated to assess the severity and predict outcomes in ARDS patients [18].

The two scores, assessed on the initial CXRs executed at ED presentation, were found to be independent outcome predictors in multivariate regression models including age, sex, and comorbidities; patients with higher Qure AI (cutoff 30) and RALE scores (cutoff 12) were more likely to become critical and have a fatal outcome. Other independent predictors of an adverse outcome were older age, male sex, coronary artery disease, COPD, and neurodegenerative disease. A correlation between higher baseline CXR scores and lower PaO2/FiO2 was also observed. Hazard ratios of the two radiological scores were similar and mostly higher than those of the clinical risk factors. Our results confirm those of Toussie et al, who have previously validated the use of initial CXR severity scores as independent outcome predictors, but on a larger population which included older patients and a longer follow-up (at least 25 days) with a significant number of negative outcome events [13]. Lung disease severity, assessed on the CXR at ED presentation, represents a valuable prognostic factor, which should be taken into consideration by medical teams in triage decisions.

In addition to this, we evaluated the use of an AI system to predict outcomes in patients with COVID-19. The comparable performance of the AI system with respect to a radiologist-assessed score in predicting adverse outcomes could represent a game-changer for resource-constrained settings as the COVID-19 pandemic keeps spreading, especially for those countries with a shortage of radiological expertise. The possibility of having the lung disease severity rapidly assessed by an AI system, together with patients’ clinical data, may help medical teams identify patients at a higher risk of an adverse outcome straight at the ED presentation and thus allocate the limited resources more efficiently.

The main limitation of this study is the retrospective design, which can lead to observer bias. Another limitation is that this single-center study was conducted in one of the hospitals in the frontline dealing with the COVID-19 outbreak in the Lombardy region (Italy) which put the local health system under severe strain forcing indications for hospital access. Presentation to EDs was instructed only for those with moderate-severe clinical conditions while patients with mild symptoms were instructed to remain at home. This could explain the high CXR’s sensitivity in our cohort (79.6% and 86.9% respectively for the AI system and radiologists’ assessment). Furthermore, the decision to group patients in the combined outcome critical COVID-19, which included all patients admitted in ICU (*n* = 80) and those who died prior to being transferred to ICU (*n* = 99), may have led to an overlap between the outcomes. Nevertheless, the scores obtained on CXRs at admission were able to predict mortality of SARS-CoV2-positive patients and can be used as repeatable, accurate, and defined tools to stratify patients and predict outcomes upon presentation. In addition, the results of this retrospective analysis have to be proven and verified prospectively in a larger population which should include patients with mild disease. Lastly, this study considered only the initial CXR analysis; further studies with sequential CXRs analysis are required to understand the disease progression in relation to therapeutic response.

## Conclusions

Our study has shown that initial CXR’s severity assessed by a deep learning AI system may have prognostic value in COVID-19 patients, with a performance comparable to a radiologist-assessed score. A Qure AI score ≥ 30 or a RALE score ≥ 12 on the CXR at ED presentation were independent and comparable predictors of adverse outcomes. We suggest that lung disease severity at ED presentation, as seen as opacification on the initial CXR, should be considered in the risk-stratification of COVID-19 patients, especially in resource-constrained settings.

## Abbreviations

AI: Artificial intelligence
AUC: Area-under-the-curve
COVID-19: Coronavirus disease 2019
EC: Ethics committee
ED: Emergency department
HR: Hazard ratio
RALE: Radiographic Assessment of Lung Edema score
ROC: Receiver operating characteristic curve
RT-PCR: Real-time reverse transcriptase polymerase chain reaction
SARS-CoV-2: Severe acute respiratory syndrome coronavirus 2
